# PAIN, PLACE OF DEATH, AND USE OF PAIN TREATMENT AT THE END OF LIFE: URBAN–RURAL DIFFERENCE AMONG CHINESE OLDER ADULTS

**DOI:** 10.1093/geroni/igae098.1745

**Published:** 2024-12-31

**Authors:** Yaolin Pei, Yifan Lou, Xiang Qi, Zheng Zhu, Jing Wang, Bei Wu

**Affiliations:** University of Texas at Austin, Texas, United States; Virginia Commonwealth University, New Haven, Virginia, United States; New York University, New York, New York, United States; New York University, New York, New York, United States; University of New Hampshire, Durham, New Hampshire, United States; New York University, New York, New York, United States

## Abstract

Pain is a common symptom that older adults encounter at the end of life and has a negative impact on quality of life. The purpose of this study was to investigate the prevalence of pain and the association between pain, place of death, and pain treatment in the context of an urban-rural division. The sample derived from the end -of-life module data from the China Longitudinal Aging Social Survey conducted in 2020. The working sample included 958 decedents aged 60 and above. Logistic regression models were used in this study. 26.4% of the decedents did not receive prompt and effective pain treatment. 66.50% of decedents’ families were not able to control the pain for the decedent. 41.96% of the decedents had severe pain symptoms. Older adults who were rural residents, had no severe pain symptoms, and died at home were more likely not to receive timely and effective pain treatment than their counterparts. The urban -rural differences in these associations were identified: Rural older adults who had severe pain symptoms and die at home were more likely not receive timely and effective pain treatment than urban older adults. Severe pain symptoms and death place were significantly associated with whether receive timely and effective pain treatment among urban older adults, but not significant among rural older adults. It is important to strengthen pain management and bridge the gaps in this regard for older adults at the end of life in China.

